# Long-Term Quantitative Biodistribution and Side Effects of Human Mesenchymal Stem Cells (hMSCs) Engraftment in NOD/SCID Mice following Irradiation

**DOI:** 10.1155/2014/939275

**Published:** 2014-02-11

**Authors:** Sabine François, Benoit Usunier, Luc Douay, Marc Benderitter, Alain Chapel

**Affiliations:** ^1^PRP-HOM, SRBE, Laboratory of Radiopathology and Experimental Therapy, Radiological Protection and Human Health Division, Institute of Radiological Protection and Nuclear Safety, 92260 Fontenay-aux-Roses, France; ^2^UMRS 938 Department of Hematology, Saint Antoine Hospital APHP and UPMC University, 75012 Paris, France

## Abstract

There is little information on the fate of infused mesenchymal stem cells (MSCs) and long-term side effects after irradiation exposure. We addressed these questions using human MSCs (hMSCs) intravenously infused to nonobese diabetic/severe combined immunodeficient (NOD/SCID) mice submitted to total body irradiation (TBI) or local irradiation (abdominal or leg irradiation). The animals were sacrificed 3 to 120 days after irradiation and the quantitative and spatial distribution of hMSCs were studied by polymerase chain reaction (PCR). Following their infusion into nonirradiated animals, hMSCs homed to various tissues. Engraftment depended on the dose of irradiation and the area exposed. Total body irradiation induced an increased hMSC engraftment level compared to nonirradiated mice, while local irradiations increased hMSC engraftment locally in the area of irradiation. Long-term engraftment of systemically administered hMSCs in NOD/SCID mice increased significantly in response to tissue injuries produced by local or total body irradiation until 2 weeks then slowly decreased depending on organs and the configuration of irradiation. In all cases, no tissue abnormality or abnormal hMSCs proliferation was observed at 120 days after irradiation. This work supports the safe and efficient use of MSCs by injection as an alternative approach in the short- and long-term treatment of severe complications after radiotherapy for patients refractory to conventional treatments.

## 1. Introduction

Multipotent stromal cells, also named mesenchymal stromal cells or mesenchymal stem cells (MSCs), have been identified in the bone marrow as multipotent progenitor cells that differentiate into osteocytes, chondrocytes, adipocytes, and stromal cells [1, 2]. In human, the use of MSCs is being tested for tissue remodelling including cardiovascular repair, treatment of lung fibrosis, spinal cord injury, and bone and cartilage repair; for review see [3, 4]. Regarding radio-induced lesion studies showed engraftment of MSCs at the site of injury [5–7] and repair to damaged tissues [8–11] but no one evaluated the long-term MSC engraftment. MSCs biodistribution has been studied in animal [12]; however, only one report described human MSC (hMSC) engraftment in human. The amount of MSCs detected in most tissues is exceedingly low and transitory. A low level of MSCs donor DNA was identified in bone marrow in some patients [13, 14]. Von Bahr et al. examined autopsies and tissue samples of patients treated with MSC infusions to assess MSC engraftment and examine the risk of tumour and/or ectopic tissue formation. Among the fifteen patients available for PCR analysis, long-term engraftment of MSCs appeared to be low due to rejection by the recipient immune system or an inability of MSCs to survive and engraft after intravenous infusion. MSC injections did not induce side effects [15]. Finally, in spite of successful clinical trials using MSCs, there is few data concerning the long-term engraftment and side effects after MSC systemic injections; transplanted stem cells must not form teratomas or undergo transformation and they must not promote tumour recurrence.

Clinical trials using MSCs in accidental irradiation [16], late severe damage of irradiation [17, 18], or radiotherapy [19, 20] of breast cancer allowed for tissue regeneration when other therapies failed [21]. There is more to consider than radiation induced DNA damage and long-term inhibition of growth of exposed cells mediated by P53 and other pathways. P53 activation stops cell growth long enough to repair the DNA damage. MSC therapy allows cells to continue to repair the tissue over the short term [22]. The long-term consequences of this “repair” are not known. Allowing the cells to progress through cell cycle with a damaged DNA template will result in severe long-term consequences including cancer induction. In this report we have investigated the long-term effect of hMSCs injected just after irradiation. We describe a xenogeneic experimental transplant model that we built to evaluate the full long-term potential of hMSC engraftment after intravenous injection and to examine the risk of tumour in irradiated tissues and ectopic tissue formation. We have tested at long-term period after irradiation (120 days) whether MSCs engraftment depends of the configuration irradiation and could induce any abnormalities, neoplastic tissue, or uncontrolled cell proliferation.

We used NOD/SCID mice to evaluate the engraftment of hMSCs in irradiated tissues. Our results showed that local irradiation increased engraftment with a direct correlation with the dose of irradiation and modified the biodistribution of hMSCs in tissues. We observed a long-term engraftment in some organs and no side effects of hMSCs over a long-time period in irradiated tissues. We believe that these observations are relevant to several clinical situations such as total body irradiation given as a pretransplant conditioning regimen, radiotherapy for the treatment of cancer, and accidental irradiation, in promoting the use of MSC infusion as part of the therapeutic scheme for the treatment of radiation side effects after radiotherapy in the short and long term.

## 2. Materials and Methods

*Isolation, Purification, and Expansion of hMSCs*. Bone marrow cells were obtained from iliac crest aspirates from healthy volunteers after informed consent and were used in accordance with the procedures approved by the Human Experimentation and Ethic Committees of Hospital St Antoine as previously described [6, 7].

*hMSC Infusion into NOD/SCID Mouse Model*. All experiments and procedures were carried out in accordance with the Guide for the Care and Use of Laboratory Animals as published by the US National Institutes of Health (NIH Publications Numbers 85-23, revised 1996), with European Directives (86/609/CEE), in compliance with the French Ministry of Agriculture Regulations for Animal Experimentation (Act reference 87-847, October 19, 1987, modified May 2001), and approved by the Local Ethical Committee (P04-07). NOD-LtSz-scid/scid (NOD/SCID) mice from breeding pairs originally purchased from Jackson Laboratory (Bar Harbor, ME, USA) were bred in our pathogen-free unit and maintained in sterile microisolator cages. A total of 134 eight-week-old male mice, divided in 4 groups, were used for this study. Group 1 was not irradiated before receiving 2.5 millions of hMSC intravenously (10 mice). Group 2 received local irradiation to the right posterior leg from 15 to 25 Gray (28 mice). Group 3 received local irradiation to the abdomen at a dose of 8.5 Gray (36 mice). Group 4 received TBI at a sublethal dose of 3.5 Gray (50 mice). Irradiations were performed as previously described [6]. Groups 2, 3, and 4 received intravenous hMSC infusion, 24 hours after irradiation. Animals were sacrificed from 3 to 120 days after irradiation and the quantitative and spatial distribution of the hMSC was studied through PCR and immunohistological analyses. Peripheral blood, bone marrow (femur), heart, lungs, liver, kidneys, spleen, stomach, gut, brain, right leg, quadriceps muscles, tibias, and skin were collected. Second-passage hMSCs were collected for infusion; the rates of viability to blue trypan were of 98%.

*Detection and Quantitative Analysis of Engrafted hMSCs, DNA Extraction, and PCR Analysis*. Human cells (hMSCs) in mouse tissues were detected as previously described by François et al. The biological samples were submitted to DNA extraction and PCR analysis to detect the presence of human cells in mouse recipients. Genomic DNA for PCR analysis was prepared from tissues using the QIAamp DNA Mini Kit, Qiagen. Amplification of human beta-globin gene was used to quantify the amount of human DNA in each sample of mouse tissue after DNA extraction. Endogenous mouse RAPSYN gene (Receptor-Associated Protein at the Synapse) was also amplified, as an internal control. Absolute standard curves were generated for the human beta-globin and mouse RAPSYN genes. Evaluation of human specificity of human beta-globin amplification was demonstrated using tenfold dilution between 100 ng and 0.05 ng of hMSC DNA with mouse DNA, without cross-reactivity, to quantify human cells in mouse tissue. One hundred nanograms of purified DNA from various tissues was amplified using TaqMan universal PCR master mix (Applied Biosytems). The primers and probe for human beta-globin were forward primer 5′GTGCACCTGACTCCTGAGGAGA3′ and reverse primer 5′CCTTGATACCAACCTGCCCAGG3′; the probe labelled with fluorescent reporter and quencher was 5′FAM-AAGGTGAACGTGGATGAAGTTGGTGG-TAMRA-3′. The primers and probe for mouse RAPSYN gene were forward primer 5′ACCCACCCATCCTGCAAAT3′ and reverse primer 5′ACCTGTCCGTGCTGCAGAA3′; the probe labelled with fluorescent reporter and quencher was 5′FAM-CGGTGCCAGTGATGAGGTTGGTC-TAMRA-3′.

### 2.1. Immunohistochemistry

To assess the tissue architectural changes induced by exposure to ionizing radiation, we performed a histological study of all organs collected and analyzed by PCR.

Before each sampling, the NOD/SCID mice were anesthetized by an intraperitoneal injection of 350 of Imlagen/Rompun solution (350 *μ*L). Blood is collected directly by intracardiac punction and the animals were perfused with saline solution to remove blood from the organs. Each organ is divided into two parts: one that will be incubated 12 hours in a solution of 4% paraformaldehyde (for histological study) and the other part will be stored in 1 mL of RNAlater buffer at −20°C (for PCR analysis).

One half of each tissue was used for PCR and immunohistology. For histological study, samples were fixed for 12 hours in 4% paraformaldehyde.

Following paraformaldehyde fixation, the organs were rinsed with distilled water and dehydrated. Tissues were sectioned at 5 *μ*m on a rotary microtome (LEICA). For immunohistochemical staining of the paraffin embedded samples, microtomed sections were deparaffinized in xylene and rehydrated though ethanol baths and PBS 1x. The sections were dip into PBS-triton (0.1x) in order to increase tissue permeability. After being rinsed with distilled water, the sections were digested with 2% trypsin for 30 minutes resulting in the endogenous biotin being blocked. The polyclonal anti-beta-2-microglobulin antibody (product NCL-B2Mp, Novocastra) was added at a dilution of 1 : 50. Negative controls were incubated with rabbit IgG diluted to 1 : 100. Detection of bound primary antibody was carried out with biotinylated secondary antibody. The biotinylated anti-rabbit IgG secondary antibody was diluted to 1 : 200 in PBS 1x and incubated for 8 minutes. The immunoreactivity was performed using alkaline phosphatase reaction with a FARED substrate detection kit for 30 minutes. For antibody detection we used the Ventana kit, followed by counterstaining with hemalun for 4 minutes. This procedure was performed using NexES special staining automation system to ensure reproducibility. On successive sections we carried out a hematoxylin-eosin-saffron (HES) staining of paraffined slides that was performed on all tissues collected at 3 and 15 days after TBI. For a global vision of morphology and tissue damage, the observation of HES-stained section (5 *μ*m) of splenic biopsies has been realized by using normal brightfield microscopy. Every 25 *μ*m, one section is colored by HES staining. In general, 5 or 7 sections of tissue were analyzed for each animal. Each section was observed by microscopy in its entirety with objective ×20. The cell depletion is determined by the decrease of blue purple chromatin intensity. Color intensity could be quantified by Histolab software. All spleens (10 per group) were collected and analyzed by microscopy. All HES-stained sections have been performed at the same time to avoid seeing the differences in color intensity. The chromatin intensity is measured with 5 random areas per tissue section.

## 3. Results

hMSCs implanted in mouse tissues were detected by PCR of the for human *β*-gene globin. Human DNA accounted for engraftment. Results are expressed as the number of mice (or percentage) with an hMSC implantation in an organ (PCR positive for the gene for human *β*-globin) in comparison to the total number of mice injected with hMSCs. Day 0 the hMSC biodistribution kinetics corresponds to the day of irradiation. hMSCs were intravenously injected 24 hours after irradiation. The control (named sham) consists in nonirradiated mice injected with 5 · 10^6^ hMSCs.

*Infused hMSCs Are Present for a Short-Time Period in Nonirradiated NOD/SCID Mice Tissues (Table 1)*. A preliminary experiment was conducted to determine in which organs hMSCs home in nonirradiated animals. The rate of implantation of human cells was determined on a series of nonirradiated mice injected with 5 · 10^6^ hMSCs (sham animals). The percentages represent the ratio of positive mice for one organ for the presence of human *β*-globin over the total number of mice analyzed. Human DNA (PCR analysis) accounted for engraftment of hMSCs following their infusion into nonirradiated animals in various tissues. The qualitative study of MSC engraftment in sham mice is shown in Table 1. Three days after injection, without irradiation, hMSCs isolated from human bone marrow migrated to murine bone marrow (10 mice PCR positive for the gene for human *β*-globin on 10 mice, 100%). Without irradiation, lung, brain, liver, and spleen appeared to be a preferential site of migration for the injected human cells. Human cells were detected only in 20% of animals whose peripheral blood was analyzed.

*hMSC Engraftment Is Related to the Irradiation Dose (Table 2)*. The biodistribution was investigated in function of the dose of irradiation 6 weeks after a local irradiation of the right posterior leg at 15 to 25 Gy.

hMSCs appeared to migrate preferentially in the organs of the irradiated area; their implantation rate seemed to be dose-dependent in these organs. For example, in bone, the percentage of hMSC engraftment varied from 12.5% at 15 Gy to 38% at 20 Gy and finally 100% of injected mice at 25 Gy.

These human cells also migrated in organs outside of the irradiated area such as kidneys. The site and frequency of implantation seem to be dose-dependent.

**Figure 1:**
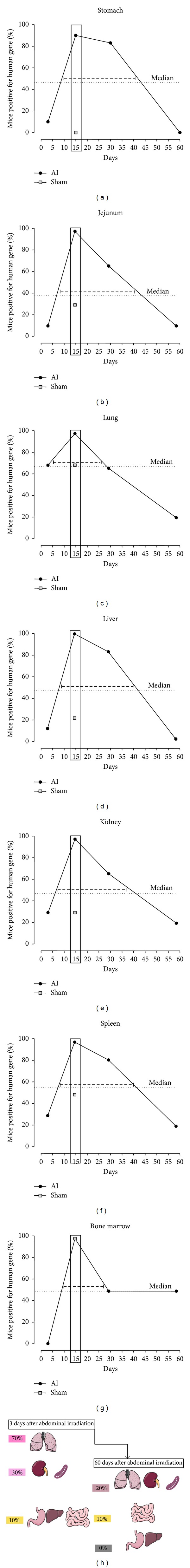
Kinetics of implantation of hMSCs during 60 days after abdominal irradiation. The percentage of positive mice for the human *β*-globin gene detected by PCR (AI) has been calculated after abdominal irradiation (AI). The human gene has been detected in (a) stomach, (b) jejunum, (c) lungs, (d) liver, (e) kidney, (f) spleen, and (g) bone marrow. The tissues were collected at 3, 15, 30, and 60 days after acute AI. The percentages represent the ratio (number of positive mice for the human beta-globin presence divided by total number of animals tested). At each analysis time, each group consisted of 10 animals. The control (named sham) consists in nonirradiated mice injected with 5 · 10^6^ hMSCs. The dotted line represents the median of the positive animal percentage during the 60 days after radiation exposure. (h) Illustration of anatomical variations in the presence of MSC after acute abdominal radiation.

*Configurations of Irradiation Modify Long-Term Biodistribution of hMSCs in Organs (Table 3, Figure 1)*. Table 3 summarizes the levels of hMSC engraftment after abdominal irradiation. Mice received localized irradiation to the abdomen. Sixty days after abdominal irradiation, very few mice were positive for human DNA. Biodistribution of human cells is maximum at 15 days. hMSC engraftment was detectable from 3 days after irradiation to at least 60 days. hMSC engraftment varies from 12% of organs at day 3 (D3), 54% on D15, 51% on D30, to 17% at day 60. The homing of human cells appeared to increase up to day 15 or day 30 depending on organs and then decreased with time. After abdominal irradiation, hMSCs appeared to preferentially migrate into the lungs, liver, kidneys, and spleen. For these organs, more than 50% of the animals were positive for the presence of the human gene at 2 and 4 weeks after irradiation. Injected human cells also colonized the abdominal muscles, the heart, liver, stomach, jejunum, quadriceps, bone (femur), and bone marrow but more moderately. For these organs, 35 to 50% of animals were positive for the presence of hMSCs. However very few hMSCs were engrafted in other parts of the intestine and the skin; for these organs less than 20% of the animals were positive for the presence of hMSCs.

Abdominal irradiation as compared to total body irradiation (TBI) modifies hMSC engraftment in the exposed area, that is, biodistribution. Abdominal irradiation increases hMSC engraftments from 3 to 60 days in stomach, duodenum, jejunum, and ileum and decreases engraftment in cecum and colon. Conversely, neither localized irradiation nor TBI modified hMSC engraftment in lung.

*hMSC Implantation Increases during the First Two Weeks after Total Body Irradiation (TBI) and Persists for a Long-Time Period in Lung and Liver (Table 4, Figure 1)*. hMSC implantation (human DNA) was detectable in lung 24 hours after intravenous injection of human cells in lung.

Day 3 after-TBI, hMSCs were localized in heart, lung, liver, muscle (abdominal), stomach, and intestine (duodenum and jejunum, distal colon). They were not detectable in the bone marrow until day 7. hMSCs isolated from bone marrow did not seem to preferentially colonize their tissue of origin in xenogeneic mouse model.

Day 7 after-TBI, hMSCs appeared to preferentially colonize the lung (100%), spleen, stomach, and heart (75%). hMSCs were still present in muscle (50%) and distal colon (25%). HMSCs started to engraft in bone (25%) and bone marrow (12.5%).

**Figure 2:**
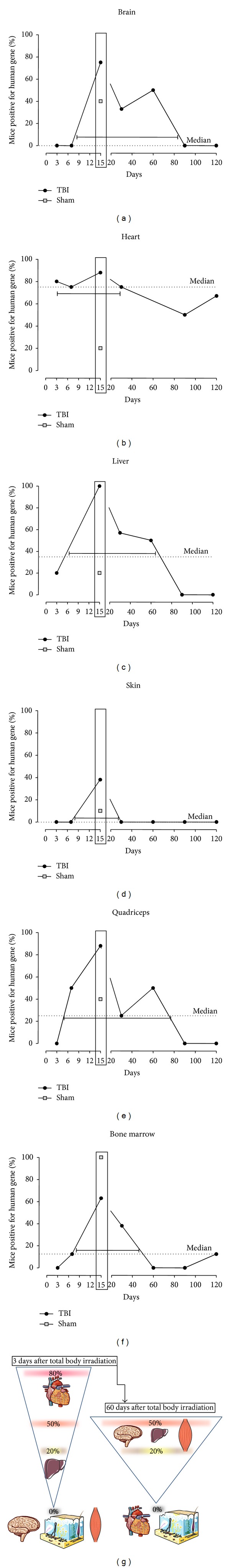
Kinetics of implantation of hMSCs during 120 days after total body irradiation. The percentage of positive mice for the human *β*-globin gene detected by PCR has been calculated after a sublethal total body irradiation (TBI). The human gene has been detected in (a) brain, (b) heart, (c) liver, (d) skin, (e) quadriceps, and (f) bone marrow. The tissues were collected at 3, 7, 15, 30, 60, and 120 days after TBI. The percentages represent the ratio (number of positive mice for the human beta-globin presence divided by total number of animals tested). At each analysis time, each group consisted of 10 animals. The control (named sham) consists in nonirradiated mice injected with hMSCs. The dotted line represents the median of the positive animal percentage during the 60 days after radiation exposure. (g) Illustration of anatomical variations in the presence of MSC after sublethal TBI.

**Figure 3:**
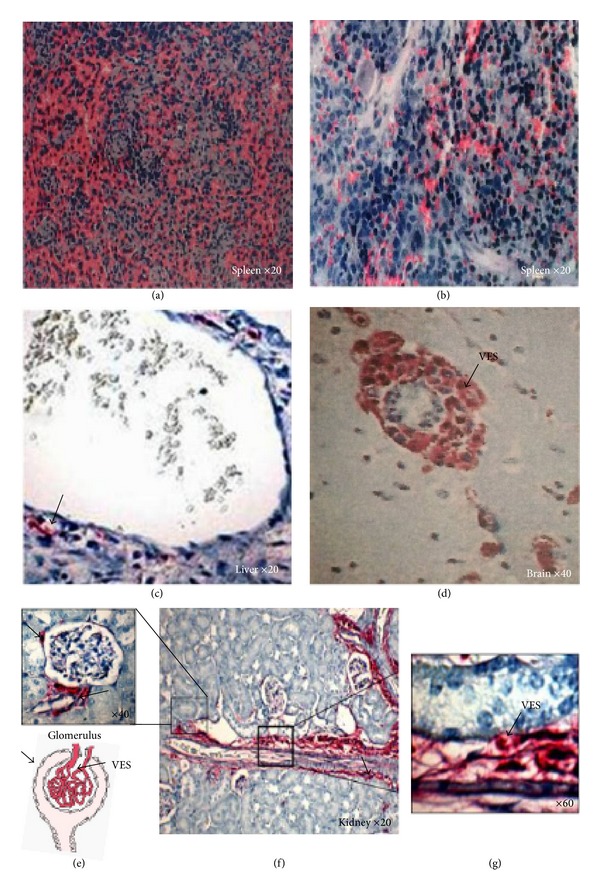
Histological examination of radiation exposed tissues by HES staining and human beta-2-microglobulin immunostaining in spleen and kidney. (a) illustrates a spleen of nonirradiated mice (control), (b) shows cell depletion 15 days after total body irradiation. The human cells expressing the human beta-2-microglobulin are stained in red. hMSCs migrated around the vascular axis (f), in functional structure such as glomerulus (e). Into glomerulus human cells have integrated both unitary structures: the glomerulus (e) and vascular endothelial system of this organ (g).

Day 15 was the short-term point at which biodistribution of hMSCs was the largest (Figure 2). The number of tissues in which the gene for human *β*-globin is present increased during the first two weeks, from 14% on day 3, 28% on day 7, to 47% on day 15. 100% of livers, 75% brain, and 50% of kidneys (see localization in Figures 2(c), 2(d), 2(e), and 2(f)) were positive for human DNA by PCR. hMSCs were implanted in all organs with the exception of the distal colon and ileum. An hMSC circulation was observed in peripheral blood (as illustrated in Figures 2(c), 2(d), and 2(e)) which corresponded to the time when the engraftment was maximum in tissues. The presence of hMSCs in peripheral blood may be due to the dissemination of cells in a large variety of tissues. At 15 days, implantation of hMSCs into muscle was detected in 88% of quadriceps. In the intestine, engraftment was observed in duodenum (33%), jejunum (25%), and proximal colon (38%). The duodenum and proximal colon were the part of the intestine most colonized by hMSCs. Very few skins and bone (femurs) analyzed were positive by PCR, 38% and 12.5% at 15 days, respectively. To localize human cells in engrafted tissues, we performed immunologic experiment using a human beta-2-microglobulin specific antibody. 14 days after hMSC graft, we observed by microscopy that human cells were integrated into functional tissue structures such as vascular endothelial system (VES) of different organs (especially in liver (Figure 3(c)), brain (Figure 3(d)), and kidney (Figures 3(f) and 3 (g))) and tissue functional units such as the glomerulus (Figure 3(e)).

**Figure 4:**
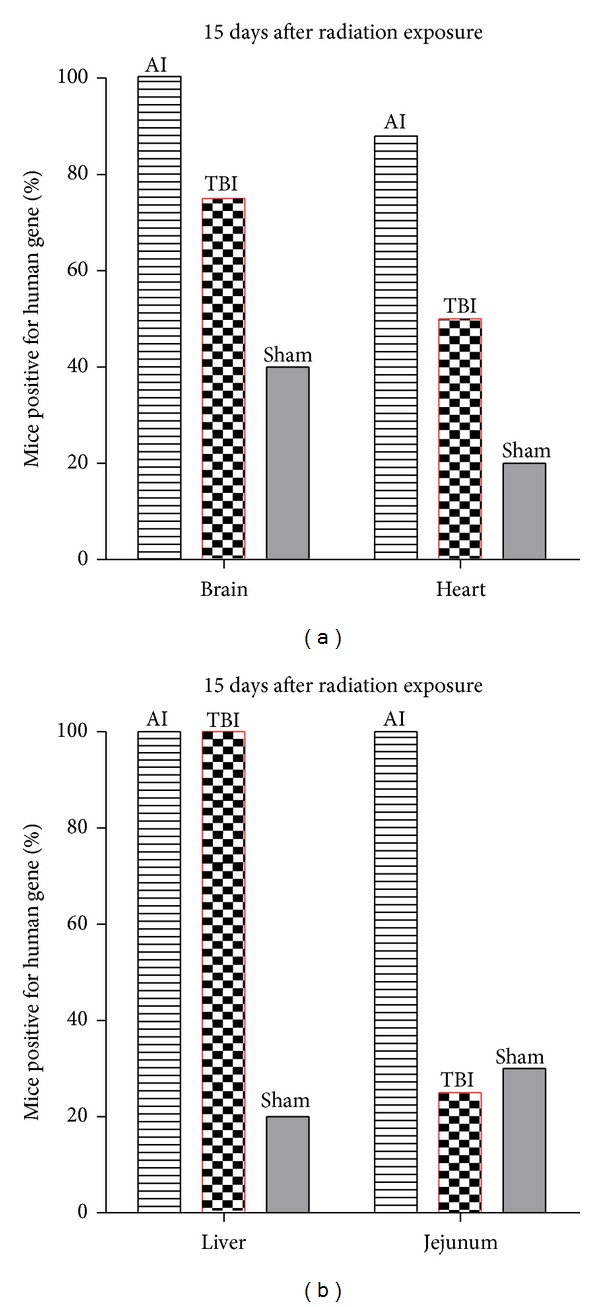
Radiation exposure promotes optimal chemotaxis of MSC. The percentage was calculated 15 days after radiation exposure and compared according to the irradiation configuration (abdominal irradiation (AI), represented in gray strips; total body irradiation (TBI) in red; and sham control group in gray). The histogram (a) represents the presence of the human beta-globin gene in organs outside the abdominal area such as the brain and heart. The histogram (b) represents the presence of the human gene in abdominal pelvic organs such as the liver and jejunum. Each group consisted in 10 animals. 15 days after AI, 100% of brain and 83% of hearts have human DNA. AI resulted in a mobilization of injected human stem cells on all the body via the vasculature system promoting communication into and between organs.

Compared to nonirradiated mice, we observed that preferential colonization sites of hMSCs appeared to be modified by radiation exposure.  Figure 4 indicates the quantitative engraftment 15 days after irradiation. TBI before hMSC infusion increased the levels of engraftment in brain, hearth, liver (Figure 4(a)) but not all in jejunum compared to nonirradiated mice (sham, Figure 4(b)). Irradiation at local site (abdomen) increased hMSCs implantation not only at the site of local irradiation (Figure 4(b)), but also in distant organs outside the local irradiation field (brain, heart, Figure 4(a)). This suggests mobilization induced by cytokines and potentially specific homing induced by chemokines, all released by inflammation.

At 30 days after-TBI, hMSCs were detected in all types of tissues except the duodenum, ileum, and the skin. Nevertheless, the hMSC biodistribution was more important at D15 (47%) than D30 (34%). After D30, the biodistribution of hMSCs injected was reduced. The number of positive blood analyzed by PCR increased at D30 (57%), suggesting a recirculation of human cells after implantation in the organs. In the intestine, hMSCs were present, in 25% of jejunum, 25% of ileum, 38% of cecum, 43% of proximal colons, and 20% of distal colons analyzed. hMSCs were not detected in the ileum and duodenums analyzed. Over a longer-time period, the duodenum was not the most colonized part of the intestine by MSCs. Although the intestine is a tissue with a rapid renewal, MSCs appeared to have incorporated all other sections of the intestine several months after-injection.

The time in which the implantation of hMSCs was optimum varied from one organ to another (Figure 1). The movement of hMSCs in the peripheral blood was maximum at day 30. For stomach, duodenum, and spleen, the highest hMSC implantation was observed at day 7. For bone marrow, muscle, brain, kidneys, liver, lungs, heart, jejunum, and cecum, after TBI, the maximum of hMSC engraftment in mice corresponds to day 15. For bone and skin, the maximum engraftment of hMSCs appeared later at days 60 and 90, respectively. The implantation of human cells appeared to decrease with time between D30 and D90.

hMSCs were detectable at least up to 120 days after injection of human cells in a number of organs. In the long term after TBI, hMSCs preferentially stayed in lung (100%), heart (67%), kidney (67%), muscle (33%), cecum and distal colon (33%), and stomach and bone marrow (17%).

*hMSCs Do Not Induce Tissue Abnormality in Irradiated Tissues after Long-Term Injection (Table 5)*. The trichromatic staining HES was performed on tissue taken between day 3 and day 120 after-irradiation. Histological study on D15 has highlighted that irradiation induced cell depletion in spleen (Figures 1(a) and 1(b)). 120 days after total body irradiation, histological observation of all tissues was done. No abnormality was observed in mice injected with hMSCs. hMSCs did not seem to have anarchical proliferation or any other side effect on tissue structure after long-term engraftment in irradiated mice (see Table 5). After hMSC injections, transplanted stem cells not form teratomas or undergo transformation and did not promote tumour growth. Histological analysis of various radiation-induced tissue damage was performed on all organs collected (heart, lung, liver, kidney, spleen, stomach, whole intestine, muscle and skin of the posterior femur of right leg, and the brain). Long-term histological analysis performed on different parts of the intestine (duodenum, jejunum, ileum, cecum, proximal, and distal colon) from day 3 to day 120 after irradiation revealed that the gut tissue retained its integrity in the early time after total body irradiation. No tumours were found in tissues (defined by neoplastic tissue or uncontrolled cell proliferation). Tissue samples showed no disruption, as defined by tissue discontinuity (e.g., rupture in intestinal epithelial barrier). We did not observe any abnormalities (e.g., inflammatory foci or abnormal cell proliferation). In addition, the size of the intestinal villi had not changed. No tissue damage or structural changes were observed in other organs, 120 days after TBI. Similar results were observed after local irradiation (data is not shown).

## 4. Discussion

We addressed the question of the potential therapeutic impact of MSC infusion and their potential side effects on tissues in the context of irradiation damage. We investigated the short- and long-term biodistribution of MSCs when infused intravenously (IV) to various tissues in relation to the dose after irradiation. To answer this question, we built a preclinical model in which hMSCs were infused to NOD/SCID mice, without previous irradiation, and following irradiation. Irradiation consisted of local irradiation exposure to the leg (15 to 25 Gray) or abdomen (8.5 Gray) or total body irradiation (3.5 Gray). These configurations induced damage to the exposed tissues as previously reported [6, 7]. Abdominal irradiation induced transient gut damage [9–11] and leg irradiation induced strong ulceration of the skin and muscles [8]. To assess the impact of localized irradiation which induced local damage, one group was subjected to irradiation of the right posterior leg and another group was subjected to irradiation of the abdomen.

In the described experiments following irradiation and hMSC infusion, most of the implanted human cells were found in weakly damaged areas and the frequency of engraftment of hMSCs was proportional to the dose of irradiation. This dose-dependent localization of hMSCs throughout the body after a localized irradiation suggested both unrestricted colonization of hMSCs and the existence of a global body's reaction following radiation exposure with an increased engraftment related to the dose of irradiation.

MSC migration reported here is in agreement with previous reports in radiation-induced multiorgan failure, ischemic brain injury, myocardial infarction, and acute renal failure [12]. In a different set of experiments that we conducted in a nonhuman primate model submitted to mixed gamma-neutron irradiation and infused with green fluorescent protein (GFP) labelled nonhuman primate MSCs, we observed that MSCs engrafted preferentially in regenerating tissues [5]. In NOD/SCID mice, as previously observed, two weeks after combined irradiation, hMSCs migrated to damage area [6, 7]. These results suggest that MSCs may participate in the preservation of the targeted tissues without side effects on irradiated tissues. We have previously published that MSC engraftment in irradiated tissues improves their functional recovery [7–11].

To our knowledge, no study reports the circulation of hMSCs in peripheral blood. We detected the circulation of hMSCs in peripheral blood and their passage through endothelial walls from days 15 to 30 after irradiation. The *in vivo* homing potential of hMSCs circulating in the bloodstream to the sites of injury/inflammation can be regulated by adhesion of hMSCs to the endothelium. Crossing of the endothelial barrier is another critical step in tissue migration of circulating cells [23].

Radiotherapy is used to treat 50% of cancer; 5% to 10% of patients develop late complications that alter quality of life [17]. Conventional therapies are palliative, poorly tolerated, costly, and lacking efficacy. The benefit of cell therapy by injection of mesenchymal stem cells for treatment of pelvic diseases has been documented [17]. A proof of concept was performed on 4 patients accidentally overirradiated after radiotherapy treatment for prostate cancer who suffered from chronic and fistulising colitis [18]. MSC injections might provide an efficient, safe, and well-tolerated alternative approach in the treatment of severe complications after pelvic radiotherapy for patients refractory to conventional therapy. Nevertheless understanding mechanisms in which adult somatic stem cells modulate tumour growth and long-term effect of MSCs after irradiation is essential to safely develop cell therapy as a therapeutic tool to treat radiation damage. We report no long-term side effects of hMSCs in irradiated tissues. Since the first reported trial in 1995, cultured MSCs have been used in 125 registered clinical trials (registered at http://www.clinicaltrial.gov/) without any reported side effect for cell therapy treatment. Clinical data support the long-term safety of MSCs. Furthermore the followup of patients after cell therapy treatment after-radiotherapy for breast [24], bladder, or prostate cancers [25] has never revealed side effects over a long-time period. A methodical review of clinical trials examined the safety of MSCs using MEDLINE, EMBASE, and the Cochrane Central Register of Controlled Trials (to June 2011). Systematic analysis examination for adverse events related to the use of MSCs did not identify any significant safety signals other than transient fever. This report further supports the safety of cell therapy to treat the consequence of radiation exposure in healthy tissues [26].

## 5. Conclusion

This work, along with our previously published studies on MSCs, supports the larger use of hMSC infusion to repair damaged tissues in patients following accidental irradiation and to treat side effects of radiotherapy in patient refractory to conventional treatments.

## Figures and Tables

**Table 1:** hMSC engraftment in nonirradiated mice 3 days after injection.

Organs	Number of positive mice for 10 mice
Brain	10/10 (100%)
Heart	5/10 (50%)
Lung	10/10 (100%)
Liver	10/10 (100%)
Spleen	10/10 (100%)
Abdominal area	
Kidney	10/10 (100%)
Muscle	0/10 (0%)
Stomach	9/10 (90%)
Duodenum	2/10 (20%)
Jejunum	10/10 (100%)
Ileum	1/10 (10%)
Cecum	1/10 (10%)
Proximal colon	1/10 (10%)
Distal colon	1/10 (10%)
Posterior leg	
Skin	1/10 (10%)
Muscle	4/10 (40%)
Bone	3/10 (30%)
Bone marrow	10/10 (100%)
Blood	3/10 (30%)
Number of positive organs	102/190
Frequency	54%

Human DNA accounted for engraftment. hMSCs implanted in mouse tissues were detected by PCR of the gene for human *β*-globin. The rate of implantation of human cells was determined on a series of non-irradiated mice grafted with hMSC injection at 5·10^6^ cells per mouse. The percentages represent the ratio of positive mice for the presence of human *β*-globin on the total number of mice analyzed.

**Table 2:** hMSC biodistribution in function of the irradiation dose 6 weeks after local irradiation of the right posterior leg at 15 to 25 Gy.

Organs (positive per mice)	Dose of irradiation		
	15 Gy	20 Gy	25 Gy
Brain	0/8 (0%)	4/10 (40%)	8/8 (100%)
Heart	6/8 (75%)	0/6 (0%)	8/8 (100%)
Lung	2/6 (33%)	4/10 (40%)	8/8 (100%)
Liver	0/8 (0%)	6/8 (75%)	8/8 (100%)
Spleen	0/8 (0%)	5/10 (50%)	8/8 (100%)
Abdominal area			
Kidney	2/6 (33%)	6/10 (60%)	8/8 (100%)
Muscle	2/8 (25%)	0/6 (0%)	6/8 (75%)
Stomach	0/8 (0%)	6/10 (60%)	8/8 (100%)
Duodenum	0/8 (0%)	10/10 (100%)	8/8 (100%)
Jejunum	0/6 (0%)	0/10 (0%)	6/8 (75%)
Ileum	0/8 (0%)	4/10 (40%)	6/8 (75%)
Cecum	0/8 (0%)	4/10 (40%)	6/8 (75%)
Proximal colon	0/8 (0%)	4/10 (40%)	6/8 (75%)
Colon distal	0/8 (0%)	4/10 (40%)	6/8 (75%)
Posterior leg			
Skin	0/8 (0%)	4/10 (40%	6/8 (75%)
Muscle	0/8 (0%)	1/8 (12.5%)	8/8 (100%)
Bone	1/8 (12.5%)	3/8 (38%)	8/8 (100%)
Bone marrow	2/6 (33%)	5/10 (50%)	6/8 (75%)
Number of positive organs	15/136	70/166	128/144
Frequency	11%	42%	89%

Results are expressed as the number of mice (or percentage) with hMSC implantation in an organ (PCR positive for the gene for human *β*-globin) in comparison to the total number of mice injected with hMSCs. Day 0 of the kinetics of hMSC biodistribution corresponds to the day of irradiation. hMSCs were intravenously injected 24 hours after irradiation. hMSCs appear to migrate preferentially in the organs of the irradiated area; their implantation rate seems to be dose-dependent in these organs. hMSCs were not found in blood; *n* = 10 (data are not shown).

**Table 3:** Levels of hMSC engraftment at 4 different time points after abdominal irradiation at 8.5 Gray.

Organs (positive per mice)	Days after irradiation			
	3 days	15 days	30 days	60 days
Brain	4/10 (40%)	10/10 (100%)	2/6 (33%)	1/10 (10%)
Heart	2/10 (20%)	5/10 (50%)	4/6 (67%)	4/10 (40%)
Lung	7/10 (70%)	10/10 (100%)	4/6 (67%)	2/10 (20%)
Liver	1/10 (10%)	10/10 (100%)	5/6 (83%)	0/10 (0%)
Spleen	3/10 (30%)	10/10 (100%)	5/6 (83%)	2/10 (20%)
Abdominal area				
Kidney	3/10 (30%)	10/10 (100%)	4/6 (67%)	2/10 (20%)
Muscle	1/10 (10%)	2/10 (20%)	6/6 (100%)	1/6 (17%)
Stomach	1/10 (10%)	9/10 (90%)	5/6 (83%)	0/8 (0%)
Duodenum	0/10 (0%)	2/10 (20%)	2/6 (33%)	1/10 (10%)
Jejunum	1/10 (10%)	10/10 (100%)	4/6 (67%)	1/10 (10%)
Ileum	1/10 (10%)	1/10 (10%)	2/6 (33%)	1/10 (10%)
Cecum	0/10 (0%)	1/10 (10%)	1/6 (17%)	1/10 (10%)
Proximal colon	0/10 (0%)	1/10 (10%)	2/6 (33%)	3/10 (30%)
Distal colon	0/10 (0%)	1/10 (10%)	2/6 (33%)	2/10 (20%)
Posterior leg (right)				
Skin	0/10 (0%)	1/10 (10%)	0/6 (0%)	0/10 (0%)
Muscle	1/10 (10%)	4/10 (40%)	2/6 (33%)	1/8 (12.5%)
Bone	1/10 (10%)	3/10 (30%)	5/6 (83%)	5/10 (50%)
Bone marrow	0/10 (0%)	10/10 (100%)	3/6 (50%)	4/8 (50%)
Blood	0/10 (0%)	3/10 (30%)	0/6 (0%)	0/10 (0%)
Number of positive organs	23/190	102/190	58/114	31/186
Frequency	12%	54%	51%	17%

The biodistribution of human cells is maximum at 15 days. hMSC engraftment is detectable from 3 days after irradiation to at least 60 days. hMSC engraftment varies from 12% of organs at day 3, 54% on D15, 51% on D30, to 17% at day 60. The homing of human cells appeared to increase up to day 15 and then decreased with time.

**Table 4:** Kinetics of hMSC biodistribution after total body irradiation (TBI) at 3.5 Gray.

Organs (positive per mice)	Days after irradiation						
	3 days	7 days	15 days	30 days	60 days	90 days	120 days
Brain	0/10 (0%)	0/10 (0%)	6/8 (75%)	2/6 (33%)	4/8 (50%)	0/8 (0%)	0/6 (0%)
Heart	8/10 (80%)	6/8 (75%)	7/8 (88%)	6/8 (75%)	0/8 (0%)	4/8 (50%)	4/6 (67%)
Lung	4/10 (40%)	10/10 (100%)	8/8 (100%)	6/8 (75%)	0/8 (0%)	3/8 (38%)	6/6 (100%)
Liver	2/10 (20%)	0/10 (0%)	8/8 (100%)	4/7 (57%)	4/8 (50%)	0/8 (0%)	0/6 (0%)
Spleen	0/10 (0%)	6/8 (75%)	5/8 (63%)	4/7 (57%)	4/8 (50%)	2/6 (33%)	0/6 (0%)
Kidney	0/10 (0%)	0/10 (0%)	4/8 (50%)	3/7 (43%)	0/8 (0%)	0/8 (0%)	2/6 (33%)
Abdominal area							
Muscle	2/10 (20%)	5/10 (50%)	2/6 (33%)	0/8 (0%)	0/6 (0%)	4/8 (50%)	2/6 (33%)
Stomach	4/10 (40%)	6/8 (75%)	4/7 (57%)	4/6 (67%)	0/8 (0%)	3/8 (38%)	1/6 (17%)
Duodenum	2/10 (20%)	5/10 (50%)	2/6 (33%)	0/7 (0%)	0/8 (0%)	0/8 (0%)	0/6 (0%)
Jejunum	2/10 (20%)	0/10 (0%)	2/8 (25%)	2/8 (25%)	0/8 (0%)	2/8 (25%)	0/6 (0%)
Ileum	0/10 (0%)	0/10 (0%)	0/8 (0%)	0/8 (0%)	0/8 (0%)	0/8 (0%)	0/6 (0%)
Cecum	0/10 (0%)	0/10 (0%)	3/8 (38%)	3/8 (38%)	0/8 (0%)	2/8 (25%)	2/6 (33%)
Proximal colon	0/10 (0%)	0/10 (0%)	3/8 (38%)	3/7 (43%)	0/8 (0%)	2/8 (25%)	0/6 (0%)
Distal colon	2/10 (20%)	2/8 (25%)	0/8 (0%)	1/5 (20%)	0/8 (0%)	0/8 (0%)	2/6 (33%)
Posterior leg (right)							
Skin	0/10 (0%)	0/10 (0%)	3/8 (38%)	0/6 (0%)	0/8 (0%)	3/8 (38%)	0/6 (0%)
Muscle	0/10 (0%)	5/10 (50%)	7/8 (88%)	2/8 (25%)	4/8 (50%)	0/8 (0%)	0/6 (0%)
Bone	0/10 (0%)	2/8 (25%)	1/8 (12.5%)	0/8 (0%)	4/8 (50%)	2/8 (25%)	0/6 (0%)
Bone marrow	0/10 (0%)	1/8 (12.5%)	5/8 (63%)	3/8 (38%)	0/8 (0%)	0/8 (0%)	1/6 (17%)
Blood	0/10 (0%)	0/10 (0%)	1/8 (12.5%)	4/7 (57%)	0/8 (0%)	0/8 (0%)	0/6 (0%)
Number of positive organs	26/190	48/178	71/151	47/137	40/150	27/150	20/114
Frequency	14%	28%	47%	34%	27%	18%	18%

The time at which the implantation of hMSCs is optimum varies from one organ to another. hMSCs were detectable at least up to 120 days after injection of human cells in a number of organs. Abdominal irradiation as compared to TBI modifies hMSC engraftment in the exposed area. Abdominal irradiation increased hMSC engraftment from 3 to 60 days in the stomach, duodenum, jejunum, and ileum and decreased engraftment in cecum and colon.

**Table 5:** Histological analysis of tissue at 120 days after total body irradiation at 3.5 Gray in hMSC injected mice.

Organs	Positive organs				Negative organs			
	Number of mice	Tumour	Tissue		Number of mice	Tumour	Tissue	
			Disruption	Abnormality			Disruption	Abnormality
Brain	1	Neg.	Neg.	Neg.	9	Neg.	Neg.	Neg.
Heart	4	Neg.	Neg.	Neg.	6	Neg.	Neg.	Neg.
Lung	2	Neg.	Neg.	Neg.	8	Neg.	Neg.	Neg.
Liver	0	ND	ND	ND	10	Neg.	Neg.	Neg.
Spleen	2	Neg.	Neg.	Neg.	8	Neg.	Neg.	Neg.
Kidney	2	Neg.	Neg.	Neg.	8	Neg.	Neg.	Neg.
Abdominal area								
Muscle	1	Neg.	Neg.	Neg.	5	Neg.	Neg.	Neg.
Stomach	0	ND	ND	ND	8	Neg.	Neg.	Neg.
Duodenum	1	Neg.	Neg.	Neg.	9	Neg.	Neg.	Neg.
Jejunum	1	Neg.	Neg.	Neg.	9	Neg.	Neg.	Neg.
Ileum	1	Neg.	Neg.	Neg.	9	Neg.	Neg.	Neg.
Cecum	1	Neg.	Neg.	Neg.	9	Neg.	Neg.	Neg.
Colon proximal	3	Neg.	Neg.	Neg.	7	Neg.	Neg.	Neg.
Colon distal	2	Neg.	Neg.	Neg.	8	Neg.	Neg.	Neg.
Posterior leg (right)								
Skin	0	ND	ND	ND	10	Neg.	Neg.	Neg.
Muscle	1	Neg.	Neg.	Neg.	7	Neg.	Neg.	Neg.
Bone	5	Neg.	Neg.	Neg.	5	Neg.	Neg.	Neg.
Bone marrow	4	Neg.	Neg.	Neg.	5	Neg.	Neg.	Neg.

Histological analysis of various radiation-induced tissue damage was performed on all organs collected (heart, lung, liver, kidney, spleen, stomach, intestine in its entirety, muscle and skin of the posterior femur of left leg, and the brain). No tissue damage or structural changes were observed in the studied organs.
